# Analysis of Brain Injury Biomarker Neurofilament Light and Neurodevelopmental Outcomes and Retinopathy of Prematurity Among Preterm Infants

**DOI:** 10.1001/jamanetworkopen.2021.4138

**Published:** 2021-04-02

**Authors:** Ulrika Sjöbom, William Hellström, Chatarina Löfqvist, Anders K. Nilsson, Gerd Holmström, Ingrid Hansen Pupp, David Ley, Kaj Blennow, Henrik Zetterberg, Karin Sävman, Ann Hellström

**Affiliations:** 1 Institute of Health and Care Sciences, Sahlgrenska Academy, University of Gothenburg, Gothenburg, Sweden; 2 Section for Ophthalmology, Department of Clinical Neuroscience, Institute of Neuroscience and Physiology, Sahlgrenska Academy, University of Gothenburg, Gothenburg, Sweden; 3 Department of Pediatrics, Institute of Clinical Sciences, Sahlgrenska Academy, University of Gothenburg, Gothenburg, Sweden; 4 Unit of Ophthalmology, Department of Neuroscience, University Hospital, Uppsala, Sweden; 5 Pediatrics, Department of Clinical Sciences Lund, Lund University, Skåne University Hospital, Lund, Sweden; 6 Clinical Neurochemistry Laboratory, Sahlgrenska University Hospital, Mölndal, Sweden; 7 Department of Psychiatry and Neurochemistry, Institute of Neuroscience and Physiology, Sahlgrenska Academy, University of Gothenburg, Mölndal, Sweden; 8 Department of Neurodegenerative Disease, University College of London Institute of Neurology, London, United Kingdom; 9 UK Dementia Research Institute at University College of London, London, United Kingdom; 10 Region Västra Götaland, Department of Neonatology, The Queen Silvia Children’s Hospital, Sahlgrenska University Hospital, Gothenburg, Sweden

## Abstract

**Importance:**

Circulating levels of neurofilament light (NfL) and glial fibrillary acidic protein (GFAP) are important in the course of brain injury in adults, but longitudinal postnatal circulating levels in preterm infants have not been investigated.

**Objectives:**

To examine postnatal longitudinal serum levels of NfL and GFAP in preterm infants during the first 15 weeks of life and to explore possible associations between these biomarkers, neonatal morbidities, and neurodevelopmental outcomes at 2 years.

**Design, Setting, and Participants:**

This cohort study used data from 3 clinical studies, including 221 infants born before 32 weeks gestational age (GA) from 1999 to 2015; neurodevelopmental outcomes were evaluated in 120 infants. Data were collected at tertiary-level neonatal intensive care units in Gothenburg, Lund, and Uppsala, Sweden. Data analysis was conducted from January to October 2020.

**Exposure:**

Preterm birth.

**Main Outcomes and Measures:**

Serum NfL and GFAP levels, retinopathy of prematurity (ROP), intraventricular hemorrhage, and Bayley Scales of Infant Development II and III at 2 years of age, analyzed by multivariate logistic regression measured by odds ratio (OR), and receiver operating characteristic curve (ROC) analysis. Area under the curve (AUC) was also measured.

**Results:**

The 221 included infants (108 [48.9%] girls) had a mean (SD) GA at birth of 26.5 (2.1) weeks and a mean (SD) birth weight of 896 (301) grams. NfL levels increased after birth, remaining high during the first 4 weeks of life before declining to continuously low levels by postnatal age 12 weeks (median [range] NfL level at birth: 58.8 [11.5-1371.3] ng/L; 1 wk: 83.5 [14.1-952.2] ng/L; 4 wk: 24.4 [7.0-306.0] ng/L; 12 wk: 9.1 [3.7-57.0] ng/L). In a binary logistic regression model adjusted for GA at birth, birth weight SD score, Apgar status at 5 minutes, and mode of delivery, the NfL AUC at weeks 2 to 4 was independently associated with any ROP (OR, 4.79; 95% CI, 2.17-10.56; *P* < .001). In an exploratory analysis adjusted for GA at birth and sex, NfL AUC at weeks 2 to 4 was independently associated with unfavorable neurodevelopmental outcomes at 2 years corrected age (OR per 10-unit NfL increase, 1.07; 95% CI, 1.02-1.13; *P* = .01). Longitudinal GFAP levels were not significantly associated with neonatal morbidity or neurodevelopmental outcome.

**Conclusions and Relevance:**

In this study, high NfL levels during the first weeks of life were associated with ROP and poor neurodevelopmental outcomes at 2 years of age. Associations between NfL and later neurovascular development in infants born prematurely should be investigated further.

## Key Points

**Question:**

How do serum levels of the brain injury biomarkers neurofilament light (NfL) and glial fibrillary acidic protein (GFAP) change after preterm birth, and are these biomarkers associated with neonatal morbidities?

**Findings:**

In this cohort study of 221 infants born preterm, infants experienced a postnatal increase in NfL that continued for at least 1 month after birth. High serum levels of NfL were associated with the retinal neurovascular disease retinopathy of prematurity (ROP) and, in an exploratory analysis, associated with an unfavorable neurodevelopmental outcome at 2 years of age.

**Meaning:**

These findings suggest that serum levels of the neuronal injury biomarker NfL might be used as an early clinical biomarker of ROP development and neurodevelopmental outcome; however, this should be investigated further.

## Teaser

This cohort study examines postnatal longitudinal serum levels of neurofilament light (NfL) and glial fibrillary acidic protein (GFAP) in preterm infants during the first 15 weeks of life and explores possible associations between these biomarkers, neonatal morbidities, and neurodevelopmental outcomes at 2 years.

## Introduction

Brain injury biomarkers neurofilament light (NfL) and glial fibrillary acidic protein (GFAP) are specific peptides reflecting neuro-axonal injury and astroglia cell injury in the brain. NfL constitutes a part of the neuronal cytoskeleton and shows promise in evaluating acute and chronic severity and the course of neurodegenerative diseases, such as Alzheimer disease, Parkinson disease, and multiple sclerosis,^1,2,3^ as well as hypoxic and traumatic brain injuries.^4,5^ Data on NfL in the preterm infant are sparse. The protein has historically been measured in cerebrospinal fluid (CSF), but methodological advances now allow quantification of NfL in serum using single-molecule array (Simoa) technology, which strongly correlates with levels measured in the CSF.^6^ The brain-specific GFAP is located in the astroglia and is released after cell injury and death.^7^ GFAP exhibits clinical prognostic potential in adult brain injury, including traumatic injury and stroke,^8,9^ and neurodegenerative disorders.^10^ Elevated GFAP concentrations in neonates, in both serum and CSF, have been described in association with periventricular white matter injury, brain injury after extracorporeal membrane oxygenation, and in full-term infants with abnormal brain magnetic resonance imaging (MRI) and adverse neurodevelopmental outcome following asphyxia.^11,12,13,14,15^

An estimated 15 million babies are born before 37 weeks gestation annually.^16^ Due to rapid maternal and neonatal care improvements, the number of surviving preterm infants is increasing. Preterm infants are at high risk of brain injury with subsequent neurodevelopmental impairment. This is due, in part, to macrostructural brain injury intraventricular hemorrhage (IVH). However, the dominant underlying pathology is white matter damage (WMD) with affected glia accompanied by the neuronal-axonal unit anomalies.^17^ WMD is associated with reduced brain volumes in preterm infants, linked to gray matter disturbances,^18,19^ resulting in poor neurodevelopment.^20^ Furthermore, prematurity itself is associated with disturbances in both white matter and gray matter.^21,22,23^ In addition, preterm infants are at particular risk of other morbidities affecting the neurovascular unit, such as the prematurity-related disorder retinopathy of prematurity (ROP). ROP is a leading cause of childhood blindness associated with later neurodevelopmental impairment^24,25^ and reduced brain volumes,^26^ although this link is not fully understood. Studies have also highlighted the possibility of using the retina as a proxy for cerebral integrity.^27^ In unique longitudinal multicenter cohorts of preterm infants, we could describe, for the first time that we are aware of, the longitudinal levels of brain biomarkers NfL and GFAP following preterm birth and investigate the possible association with morbidities in the neurovascular unit as well as the eye and the brain, which are also key organs determining long-term outcome.

## Methods

### Study Populations

Infants from 3 Swedish cohorts of infants born before 32 weeks’ gestational age (GA) between 1999 and 2015 were included. The cohorts were earlier described separately.^28,29,30,31^

The ethics committees of the respective institutions approved the clinical studies in cohort 1 at the University of Gothenburg and Uppsala University. Approval was obtained from the regional ethical review board in Lund for cohort 2. Cohort 3 was approved by the Regional Ethical Board in Gothenburg and registered at ClinicalTrials.gov (NCT02760472). Infants were included after oral and written informed consent from parents or legal guardians. This study followed the Strengthening the Reporting of Observational Studies in Epidemiology (STROBE) reporting guideline.

Cohort number 1 included 84 infants born at less than 32 weeks gestation who were admitted to neonatal intensive care units (NICUs) at Queen Silvia Children’s Hospital (n = 70) and Uppsala University Hospital (n = 14) between December 1999 and April 2002. Blood samples were collected on postnatal day (PND) 1 and then once every week until 40 weeks postmenstrual age (PMA).

Cohort number 2 included 64 infants born at less than 31 weeks gestation and admitted to the NICU at Lund University Hospital between January 2005 and May 2007. Blood was collected at PND 3, 7, and 14, and then once every week until 40 weeks’ PMA.

Cohort number 3 included 90 infants born at less than 28 weeks gestation in the NICU at Queen Silvia Children’s Hospital between April 2013 and September 2015. Cohort 3 was a randomized clinical interventional study in which participants were randomized to different parenteral nutrition strategies regarding lipids, either an omega-3-enriched lipid emulsion or standard care. Blood samples were collected from the umbilical cord and at PND 1, 7, 14, and 28 and at PMA 32, 36, and 40 weeks.

**Figure 1.**
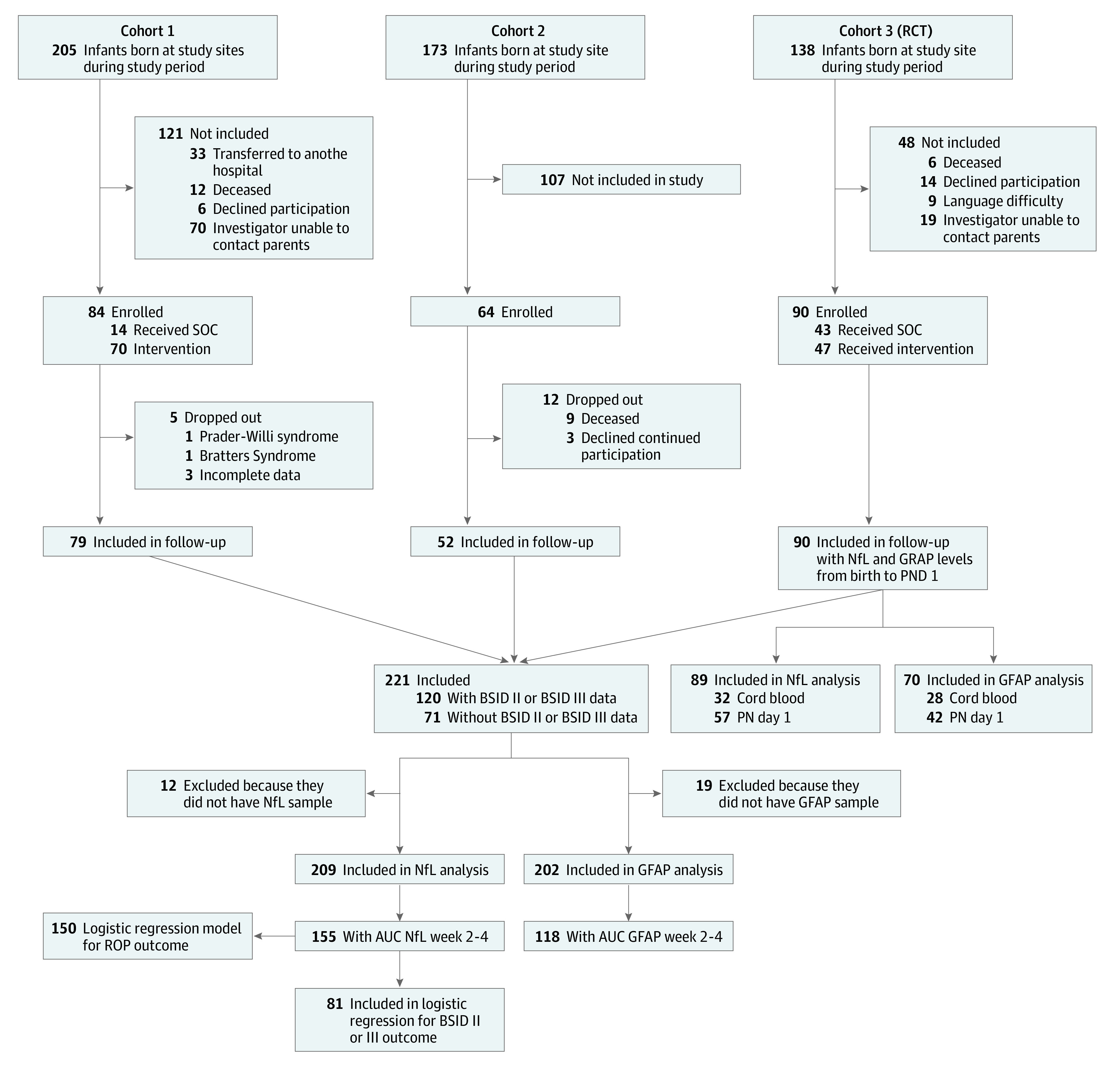
Flow Diagram of Cohort Construction AUC indicates area under the curve; BSID, Bayley Scale of Infant Development; GFAP, glial fibrillary acidic protein; NfL, neurofilament light; PN, postnatal; RCT, randomized clinical trial; ROP, retinopathy of prematurity; and SOC, standard of care.

Cohorts are summarized according to STROBE^32^ and Consolidated Standards of Reporting Trials (CONSORT) reporting guideline^33^ in Figure 1. Blood samples were analyzed from birth until 15 weeks’ PMA.

### Analytical Measurements

NfL and GFAP were measured simultaneously with all individuals randomized across the included assays using the same reagent batch of SIMOA NF-Light Advantage kit and SIMOA GFAP Discovery Kit, respectively, on the Simoa HD-X platform (Quanterix Corp). Detailed descriptions of the protocol for analytical measurements, interassay and intra-assay variability, calibration limits, and data on the number of samples analyzed are described in the eAppendix in the Supplement.

### Variable Definition

Longitudinal data on NfL and GFAP were categorized in subgroups based on the PNDs of sampling as follows: at birth, PND 0-3; week 1, PND 4-10; week 2, PND 11-17; week 3, PND 18-24; week 4, PND 25-31; week 5, PND 32-38; week 6, PND 39-45; week 7, PND 46-52; week 8, PND 53-59; week 9, PND 60-66; week 10, PND 67-73; week 11, PND 74-80; week 12, PND 81-87; week 13, PND 88-94; week 14, PND 95-101; and week 15, PND 102-108. Multiple data points during 1 of these periods from a single individual were transformed into a mean data point for the corresponding period. GFAP results are presented in the eAppendix in the Supplement, following the same statistical approach described for NfL.

Diagnoses of IVH and ROP were retrieved from clinical records. ROP was diagnosed by repeated ophthalmological examinations and defined as non-ROP or ROP (stage 1, 2, or 3). ROP evaluations were initiated according to national guidelines not earlier than at PMA 31 weeks. IVH included any stage of IVH diagnosed by cerebral ultrasound.

### Bayley Scales of Infant Development

Neurodevelopmental outcomes were assessed by Bayley Scales of Infant Development (BSID) at 2 years of corrected age in cohorts 2 (n = 48) and 3 (n = 72). Due to updated test routines between study periods, cohort 2 was evaluated by BSID II and cohort 3 by BSID III. Moderate to severe neurodevelopmental impairment in BSID II was defined as either a psychomotor developmental index (PDI) or mental development index (MDI) score of less than 70 and in BSID III as a composite cognitive, composite language, or composite motor scale score below 2 SD compared with healthy full-term infants, as retrieved from the Swedish population-based cohort Express Study.^34^ The thresholds used for BSID III closely resembled the suggested cutoff levels for moderate to severe neurodevelopmental delay in BSID III compared with BSID II.^35,36^ The categorization of neurodevelopment was based on BSID findings.

### Statistical Analysis

The identification of postnatal period of interest for further investigation was based on visual analysis of postnatal development of brain biomarkers. Statistical analyses were performed using SPSS Statistics version 26 (IBM Corp). Nonparametric statistical methods were used because NfL concentrations did not follow the normal distribution. Correlation between GA and cord blood NfL levels were investigated by Spearman correlation coefficient. The correlation between BSID scores and longitudinal NfL levels was also assessed by the Spearman correlation coefficient. To investigate the development of NfL levels after birth, the Mann-Whitney U test was used to compare concentrations at group levels with Bonferroni-Holm correction for multiple testing, and the Wilcoxon signed-rank test was used to investigate the difference in concentrations from birth (ie, cord blood) to PND 1 for infants with repeated samples. In all analyses, a 2-sided *P* < .05 was considered significant, unadjusted for multiple testing.

A measure of longitudinal NfL concentrations was calculated using the area under the curve (AUC). AUC was calculated accordingly the trapezoidal rule, as described by Fekedulegn et al,^37^ biweekly, based on NfL concentrations or linear interpolation if the value at the first or last point was not obtainable and at least 2 adjacent values were available. Cognitive and motor outcomes were used as the dependent variables in binary regression models to investigate the association between NfL AUC at weeks 2 to 4 with the neurodevelopmental outcome.

Binary logistic regression models based on significant variables were used to calculate the probability values for morbidities as odds ratios (ORs) reported with 95% CIs. In models with ROP as the outcome, the natural logarithm of NfL AUC (ln NfL AUC) was studied because of its stronger association with the outcome than that of NfL AUC (based on lower Akaike information criterion). Variables with an association of *P* < .20 for each morbidity based on the Mann-Whitney U test were considered in the models, with birth characteristics, longitudinal NfL levels, and other morbidities. Included variables were entered stepwise using the forward method. Variables significantly contributing to the models were included in the final logistic regression model with clinically relevant variables. For neurodevelopmental outcomes, the model was adjusted for GA at birth and sex.

The models were validated for linearity between the included variables and ln-odds, the independence between observations, multicollinearity, and outliers. The models did not yield any severe diagnostics when investigated for multicollinearity (variance inflation factor between 1 and 2). Goodness-of-fit for the logistic regression models was tested by the Hosmer-Lemeshow test and was found satisfactory.

Receiver operating characteristic (ROC) curves were used to evaluate the statistical associations of disease and possible cutoff values with the highest explanatory contributions to morbidities. The cutoff was determined from a table with all possible cutoff values for the coordinate with the highest sensitivity and specificity. Based on selected cutoffs, positive predictive values (PPVs) and negative predicted values (NPVs) were computed and reported with 95% CIs. The orthogonal projections to latent structures (OPLS) model is described in the eAppendix in the Supplement.

## Results

A total of 221 infants with a mean (SD) GA at birth of 26.5 (2.1) weeks and a mean (SD) birth weight (BW) of 896.5 (301.4) grams were included in the study population. The clinical characteristics of the included infants are shown in Table 1.

**Table 1.** Clinical Characteristics for the 221 Infants in Complete Study Population

Characteristics	Infants by cohort, No. (%)			
	All (N = 221)	Cohort 1 (n = 79)	Cohort 2 (n = 52)	Cohort 3 (n = 90)
GA at birth, mean (SD), wk	26.5 (2.1)	27.7 (2.2)	26.4 (1.9)	25.4 (1.4)
BW, mean (SD), g	896.5 (301.4)	1034.4 (335.6)	889.3 (284.8)	779.6 (220.6)
BW SDS, mean (SD)	−1.1 (1.4)	−1.3 (1.3)	−1.1 (1.3)	−0.9 (1.4)
SGA at birth	47 (21.3)	21 (26.5)	13 (25)	13 (14.4)
Female sex	108 (48.9)	44 (55.7)	25 (48.1)	39 (43.3)
Male sex	113 (51.1)	35 (44.3)	27 (51.9)	51 (56.7)
Twin	58 (26.2)	23 (29.1)	25 (48.1)	10 (11.1)
Mortality	12 (5.4)	Not included	Not included	12 (13.3)
Cesarean delivery^a^	136 (62.1)	50 (64.9)	44 (84.6)	42 (46.7)
Prenatal steroids^b^	187 (90.8)	57 (83.8)	50 (98.0)	80 (92.0)
Head circumference SDS, from birth to 7 PND, mean (SD)^c^	−0.5 (1.3)	−1.1 (1.3)	−0.1 (1.5)	−0.5 (1.1)
Any ROP^d^	124 (59.3)	44 (55.7)	19 (36.5)	61 (78.2)
BPD^d^	100 (47.8)	23 (29.1)	38 (73.1)	39 (50.0)
Any IVH^e^	62 (28.4)	13 (16.4)	14 (26.9)	35 (40.2)
NEC^e^	12 (5.5)	4 (5.1)	1 (1.9)	7 (8.0)

Abbreviations: BPD, bronchopulmonary dysplasia; BW, birth weight; GA, gestational age; IVH, intraventricular hemorrhage; NEC, necrotizing enterocolitis; PND, postnatal day; ROP, retinopathy of prematurity; SDS, standard deviation score; SGA, small for gestational age.

^a^ Data available for 219 infants.

^b^ Data available for 206 infants.

^c^ Data available for 180 infants.

^d^ Data available for 209 infants.

^e^ Data available for 218 infants.

### Serum Levels of NfL After Very Preterm Birth

**Figure 2.**
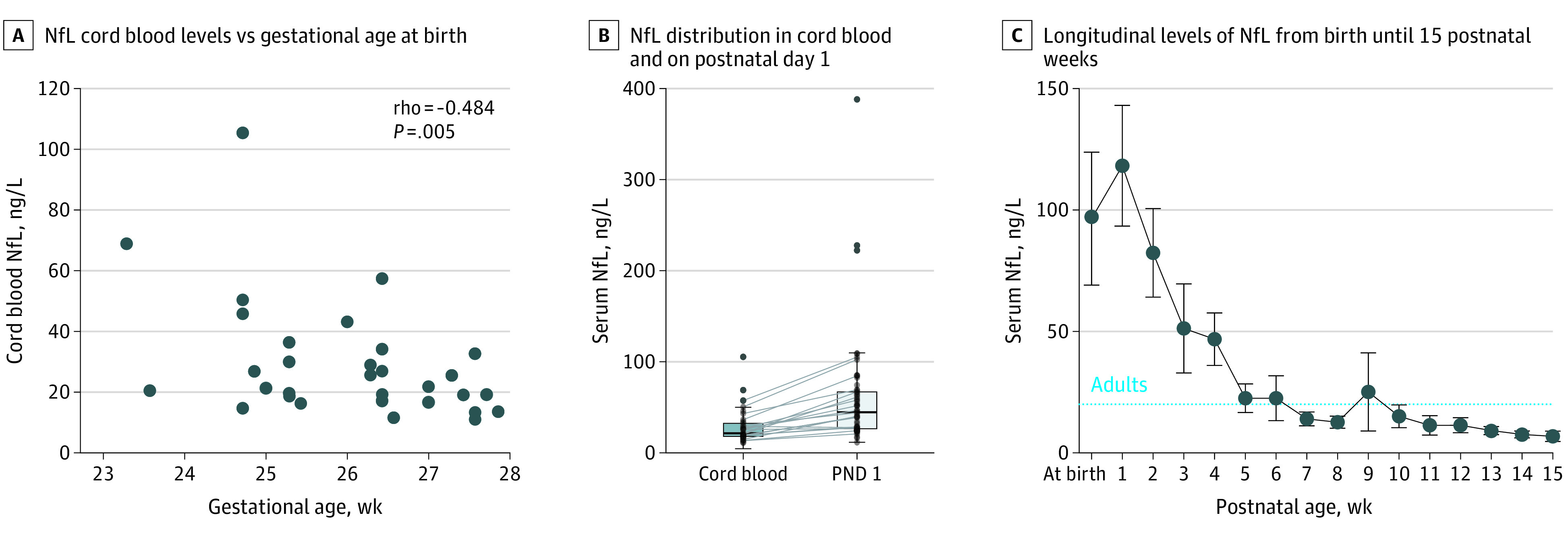
Neurofilament Light (NfL) Levels B, NfL levels significantly increased between cord blood and PND 1 samples (Wilcoxon signed-rank test, *P* < .001). Levels for 23 infants who had repeated samples are connected with lines. C, NfL was measured in 120, 128, 121, 82, 115, 98, 85, 85, 77, 77, 60, 54, 43, 43, 20, and 11 infants at birth and weeks 1-15, respectively. The dotted horizontal line represents mean levels in healthy adults.^38^ Error bars represent 95% confidence intervals.

Concentrations of serum NfL at birth (cord blood) and PND 1 were compared in a subpopulation (cohort 3). NfL cord blood levels were negatively correlated with GA (32 participants; Spearman correlation coefficient, −0.48; 95% CI, −0.72 to −0.14; *P* = .009) (Figure 2A). The median (range) NfL concentration in cord blood from 32 infants was 21.6 (11.0-105.4) ng/L and significantly greater at PND 1 among 57 infants (44.7 [11.5-438.0]; *P* < .001) (Figure 2B). Among 23 infants with both cord blood and PND 1 samples, NfL levels significantly increased during the first day of life (median [range] NfL levels at birth: 21.3 [11.6-57.4] ng/L; PND 1: 39.6 [17.4-87.8] ng/L; *P* < .001).

### Longitudinal Serum Levels of NfL and Lower GA

Longitudinal serum levels of NfL from birth to 15 weeks’ PMA are illustrated in Figure 2C. The longitudinal associations between NfL and GA for the first 12 weeks are shown in eFigure 1 in the Supplement. Infants born before 27 weeks’ GA showed a different pattern for NfL between postnatal week 1 and 10 (ie, significantly higher, except for PN week 2 and 5) than infants born after 27 weeks’ GA.

### Longitudinal NfL Levels and the Development of ROP and Other Morbidities

The biweekly period with the strongest association with IVH and ROP as well as a high number of available AUC values (n = 155) was weeks 2 to 4, and it was used for further analyses. Unadjusted, the NfL AUC at weeks 2 to 4 was higher in infants with IVH or ROP compared with infants without the respective morbidity (median [range] NfL AUC among 36 infants with vs 119 infants without IVH: 119.1 [23.3-832.2] ng/L vs 71.0 [12.2-647.9] ng/L; *P* < .001; among 92 infants with vs 62 infants without ROP: 124.9 [23.3-832.2] ng/L vs 43.3 [12.2-368.0] ng/L; *P* < .001).

**Figure 3.**
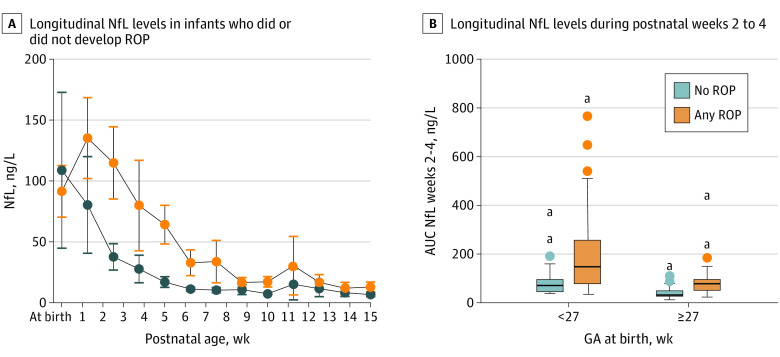
Longitudinal Levels of Neurofilament Light (NfL) Error bars indicate 95% CIs; AUC, area under the curve; GA, gestational age; ROP, retinopathy of prematurity. ^a^Extreme outlier, defined as 3 times the interquartile range.

Longitudinal levels of NfL in infants with or without ROP according to GA at birth are shown in Figure 3A and 3B. Regardless of GA at birth, infants who developed ROP had a significantly higher median (range) NfL AUC at weeks 2 to 4 than infants who did not develop ROP (<27 weeks’ GA, 75 infants with vs 18 infants without ROP; 147.8 [34.7-832.2] ng/L vs 70.9 [37.5-368.0] ng/L; *P* = .005; >27 weeks’ GA, 17 infants with vs 44 infants without ROP: 78.1 [23.3-437-4] ng/L vs 31.9 [12.2-132.7] ng/L; *P* < .001).

The ln NfL AUC at weeks 2 to 4 was tested against ROP outcome in binary logistic regression models. Ln NfL AUC at weeks 2 to 4 was independently associated with ROP adjusted for GA at birth, BW SD score, mode of delivery, and low Apgar score (ie, <7) at 5 minutes. The OR for ln NfL AUC at weeks 2 to 4 for 150 infants was 4.79 (95% CI, 2.17-10.56; *P* < .001) (eTable 1 in the Supplement). Data for model validation is available in eFigure 2 in the Supplement.

The OPLS model was used to rank clinical variables associated with NfL AUC at weeks 2 to 4. The strongest associations with NfL AUC were GA at birth, followed by BW, ventilator support for more than 7 days, and ROP (eAppendix, eTable 2, and eFigure 3 in the Supplement).

Examination by Mann-Whitney U test of variables associated with ROP yielded the significant parameters listed in eTable 3 in the Supplement. NfL concentrations were significantly higher for infants with ROP compared with no ROP regarding both weekly concentrations (weeks 2, 3, and 4) and all longitudinal AUC for the period from birth to PN week 5.

ROC analysis showed that the statistical contribution of the ln NfL AUC at weeks 2 to 4 to the assessment of the ROP outcome was higher than GA (eFigure 4 in the Supplement). The binary logistic regression model including confounding variables presented excellent explanatory value for ROP (eTable 4 in the Supplement). The identification of a single cutoff value with the highest explanatory value to ROP outcome was based on NfL concentrations between PN weeks 2 and 4 (325 available values from 171 infants). The best cutoff was found at 33.5 ng/L (eFigure 4 and eTable 4 in the Supplement).

IVH was not found to be an important variable for NfL AUC at weeks 2 to 4 based on the OPLS model (eTable 2 in the Supplement). IVH was also not associated with the NfL AUC at weeks 2 to 4 in the adjusted regression models.

### Neurodevelopmental Outcome at 2 Years of Age

Neurodevelopmental outcome was investigated in 120 infants with a mean (SD) GA at birth of 25.9 (1.7) weeks. Among 81 infants with available NfL AUC, the NfL AUC at weeks 2 to 4 was independently associated with moderate to severe neurodevelopmental impairment at 2 years, adjusted for GA at birth and sex (OR per 10-unit increase, 1.07; 95% CI, 1.02-1.13; *P* = .01). The explanatory contribution to BSID outcome was excellent (eTable 5 in the Supplement). The NfL AUC at weeks 2 to 4 had the strongest association of the included variables. Data from model validation appears in eFigure 5 of the Supplement. Furthermore, NfL AUC at weeks 2 to 4 was negatively correlated with all 5 BSID outcome measures (ie, PDI and MDI on BSID II and motor composite score, cognitive composite score, and language composite score on BSID III), as illustrated in Table 2.

**Table 2.** Correlation Between Neurofilament Light Area Under the Curve at Weeks 2 to 4 and BSID II and BSID III

Outcome	Spearman correlation coefficient (95% CI)	*P* value
BSID II		
Psychomotor developmental index	−0.33 (−0.59 to −0.01)	.04
Mental developmental index	−0.45 (−0.68 to −0.14)	.005
BSID III		
Motor composite score	−0.59 (−0.77 to −0.32)	<.001
Cognitive composite score	−0.49 (−0.70 to −0.20)	.001
Language composite score	−0.48 (−0.70 to −0.18)	.002

Abbreviation: BSID, Bayley Scales of Infant Development.

### GFAP Levels, Morbidities, and Neurodevelopmental Outcomes

Longitudinal GFAP levels were not significantly associated with neonatal morbidities or neurodevelopmental outcome. Results are presented in the eAppendix in the Supplement, with longitudinal GFAP levels in eFigure 1 and eFigure 6 in the Supplement and correlation with neurodevelopmental outcome in eTable 6 in the Supplement.

## Discussion

Very preterm birth was associated with a postnatal increase in the level of neuronal injury biomarkers NfL and GFAP, with NfL remaining at high levels for at least 1 month after birth. Increased serum levels of NfL between PN weeks 2 and 4 were independently associated with the development of the retinal neurovascular disease ROP and with neurodevelopmental outcome at 2 years of age in a subgroup of infants. Early postnatal NfL levels in the very preterm infants corresponded with levels observed after adult brain injury.^5^ At 2 months of age, levels decreased to ranges observed in healthy adults.^38^

The inverse association between the degree of immaturity and postnatal serum levels of NfL corresponds to earlier results from more mature infants; Depoorter et al^39^ also identified an association between high NfL levels on PND 1 to 7 and neonatal brain injury. Preterm infants have a higher risk of widespread microstructural anomalies and impaired functional connectivity compared with healthy full-term infants. Preterm brain development is altered even in the absence of structural brain injury.^40^ Preterm brain injury, such as IVH and WMD with associated axonal injury, is linked to subsequent neurodevelopmental disability, the latter of significant importance. We found no evident association between postnatal IVH and high NfL levels in the adjusted model. The lack of an association between IVH and NfL could be due to the inability to discriminate insults in a period characterized by heterogenic NfL concentrations. Taken together, these data indicate that NfL is more likely a biomarker of general WMD with associated axonal injury.

### Change in Serum NfL Levels Between Birth and PND 1

In a subcohort of our study group, the observed increase in serum concentrations of NfL at PND 1 compared with cord blood suggests that the transition from fetal to postnatal life is a potential insult to the developing brain. This increase in NfL levels has also been described for samples drawn at PND 3 compared with cord blood.^39^ Importantly, the observed increase in concentrations of NfL well exceeds the physiologic hemoconcentration observed immediately after birth.

### ROP, NfL, and the Retina

NfL was independently associated with ROP, and high levels were associated with poor ROP outcomes. NfL exposure during postnatal weeks 2 to 4, a critical period for vessel formation and development, had the strongest association of all included variables with ROP outcome based on ROC analysis. ROP is one of the significant severe morbidities affecting preterm infants and is associated with several aspects of abnormal neurodevelopmental outcomes, including cerebral palsy and cognitive impairment. However, this connection is not fully understood.^24,25,41,42^ ROP has also been associated with reduced brain volumes, suggesting common pathways involved in impaired neural and neurovascular development of the brain and retina.^43^ Support for the use of the retinal vasculature as a proxy for the integrity and pathology of the cerebral microvasculature has been highlighted in recent studies,^27,44^ especially for small vessel disease, as defined by white matter hyperintensities on structural MRI, visual scores, and volume; perivascular spaces; and lacunes and microbleeds. Gattringer et al^45^ also reported higher levels of NfL in adults with cerebral small vessel disease. The brain and retina are linked by vasculature and neurons. Emerging evidence has led to the classification of ROP as both a neuro- and vascular-related disease rather than just a vascular disease. Elevated serum NfL levels are also associated with retinal nerve fiber thinning in patients with multiple sclerosis.^46^ NfL has been suggested as a biomarker of neuro-axonal damage.^46^

It seems promising that serum NfL levels could be used to identify infants at risk of developing ROP. An applicable cutoff for NfL concentration was obtained between PN weeks 2 and 4; 33.5 ng/L showed promising sensitivity and specificity to determine later ROP outcomes alongside GA. However, this threshold requires prospective evaluation in larger cohorts and independent studies.

### GFAP

Apart from the immediate increase after birth, the longitudinal levels of GFAP did not indicate any distinctive pattern in relation to GA, morbidities, or other clinical variables. Both NfL and GFAP were included in a systematic review^47^ covering the usefulness of potential biomarkers for traumatic brain injury, including an evaluation of the half-life described in publications. The conclusion was that higher GFAP levels are found up to 48 hours after injury, whereas NfL levels are elevated for more extended periods following brain injury in adults.^48,49^ Speculatively, possible shifts in serum levels of GFAP would require a more elaborative blood sampling regimen with higher temporal resolution.

### NfL and Neurodevelopmental Outcome

We observed an association between NfL during the first PN weeks and long-term neurodevelopmental outcomes at 2 years, as determined by an unfavorable BSID score. This association was independent of GA at birth, previously established as a factor associated with neurocognitive outcome. A reduced BSID score at 2 years of age is associated with later adverse neurodevelopmental outcomes.^50^ Our definition of unfavorable neurodevelopment outcome was a crude measurement, flawed by the use of 2 different tests in different cohorts. However, these results may still be of interest to direct future studies. Furthermore, regardless of the various tests, NfL levels were significantly correlated with the outcome in all 5 categories individually (PDI and MDI in BSID II and motor composite score, cognitive composite score, and language composite score in BSID III).

The discovery and validation of neonatal biomarkers of brain injury is a critical step in the evolution of neonatal neuroprotection. The possibility of detecting and monitoring the effects of axonal damage and brain development in peripheral blood samples would be of tremendous benefit in clinical decision-making in neonatal intensive care.

### Limitations

This study has limitations, including its retrospective study design. In addition, the prolonged recruitment period may be a possible shortcoming, given that there have been significant developments of new clinical practices. Thus, different tests (ie, BSID II and BSID III) were used when assessing neurodevelopmental outcomes in cohorts 2 and 3. BSID III has been shown to result in higher scale points compared with BSID II, which has been the reason for attempts at conversion factors and different thresholds when combining the 2 tests.^35,51^

Another limitation is the prolonged time between sampling and biomarker measurement. NfL has been reported to be stable for as many as 5 freeze-thaw cycles and delayed freezing after blood sampling for as long as 24 hours.^52,53^ Nevertheless, the storage time is an uncertain factor that might affect the quality of the samples and may affect the measurements’ results. The fact that measurements were made on singleton samples due to the limited sample volumes increases the uncertainty in the results.

## Conclusions

In this study, preterm birth was associated with persistently increased serum levels of the brain injury biomarker NfL, corresponding to levels observed after adult brain injury. Furthermore, early postnatal increases in NfL levels were strongly associated with the neurovascular disease ROP and long-term neurodevelopmental outcomes at 2 years of age, possibly illustrating WMD with associated axonal injury. The discovery and validation of a neonatal biomarker for brain injury are crucial in neonatal neuroprotection. The promising role of NfL as a biomarker for ROP and the development of the neurovascular unit should be investigated further.

## Notes

Supplement. **eAppendix.** Analytic Measurements, Variable Definitions, Statistics, and GFAP Results **eTable 1.** Binary Logistic Regression Model for ROP Outcome **eTable 2. **Associations Between Clinical Parameters and NfL **eTable 3. **Variables With Significant Contribution to Any ROP **eTable 4. **ROC Curve Analysis for ROP Outcome **eTable 5. **ROC Curve Analysis for BSID Outcome Below the Threshold at 2 Years of Age **eTable 6. **Longitudinal GFAP Levels Summarized by AUC at Weeks 2 to 4 and Spearman Correlation to Outcome in BSID II and BSID III **eFigure 1. **Longitudinal Levels of NfL and GFAP From Birth Until 12 Postnatal Weeks **eFigure 2. **Estimated Probabilities and Calibration Plot for Binary Logistic Regression Model ROP Outcome **eFigure 3. **Permutation Test for the OPLS Model **eFigure 4. **ROC Curve Analysis for ROP Outcome **eFigure 5. **Estimated Probabilities and Calibration Plot for Binary Logistic Regression Model BSID Outcome **eFigure 6. **Longitudinal Levels of GFAP From Birth Until 12 Postnatal Weeks
