# Supplementary material for: Analysis of Brain Injury Biomarker Neurofilament Light and Neurodevelopmental Outcomes and Retinopathy of Prematurity Among Preterm Infants
Source: JAMA Netw Open. 2021 Apr 2;4(4):e214138. doi: 10.1001/jamanetworkopen.2021.4138 (PMC8019094; doi:10.1001/jamanetworkopen.2021.4138)
Supplement: Supplement. — eAppendix. Analytic Measurements, Variable Definitions, Statistics, and GFAP Results eTable 1. Binary Logistic Regression Model for ROP Outcome eTable 2. Associations Between Clinical Parameters and NfL eTable 3. Variables With Significant Contribution to Any ROP eTable 4. ROC Curve Analysis for ROP Outcome eTable 5. ROC Curve Analysis for BSID Outcome Below the Threshold at 2 Years of Age eTable 6. Longitudinal GFAP Levels Summarized by AUC at Weeks 2 to 4 and Spearman Correlation to Outcome in BSID II and BSID III eFigure 1. Longitudinal Levels of NfL and GFAP From Birth Until 12 Postnatal Weeks eFigure 2. Estimated Probabilities and Calibration Plot for Binary Logistic Regression Model ROP Outcome eFigure 3. Permutation Test for the OPLS Model eFigure 4. ROC Curve Analysis for ROP Outcome eFigure 5. Estimated Probabilities and Calibration Plot for Binary Logistic Regression Model BSID Outcome eFigure 6. Longitudinal Levels of GFAP From Birth Until 12 Postnatal Weeks [file jamanetwopen-e214138-s001.pdf]

Supplemental Online Content

Sjöbom U, Hellström W, Löfqvist C, et al. Analysis of brain injury biomarker neurofilament light and neurodevelopmental outcomes and retinopathy of prematurity among preterm infants. *JAMA Netw Open*. 2021;4(4):e214138. doi:10.1001/jamanetworkopen.2021.4138

eAppendix. Analytic Mesursements, Variable Definitions, Statistics, and GFAP Results

eTable 1. Binary Logistic Regression Model for ROP Outcome

eTable 2. Associations Between Clinical Parameters and NfL

eTable 3. Variables With Significant Contribution to Any ROP

eTable 4. ROC Curve Analysis for ROP Outcome

eTable 5. ROC Curve Analysis for BSID Outcome Below the Threshold at 2 Years of Age

eTable 6. Longitudinal GFAP Levels Summarized by AUC at Weeks 2 to 4 and Spearman Correlation to Outcome in BSID II and BSID III

eFigure 1. Longitudinal Levels of NfL and GFAP From Birth Until 12 Postnatal Weeks

eFigure 2. Estimated Probabilities and Calibration Plot for Binary Logistic Regression Model ROP Outcome

eFigure 3. Permutation Test for the OPLS Model

eFigure 4. ROC Curve Analysis for ROP Outcome

eFigure 5. Estimated Probabilities and Calibration Plot for Binary Logistic Regression Model BSID Outcome

eFigure 6. Longitudinal Levels of GFAP From Birth Until 12 Postnatal Weeks

This supplemental material has been provided by the authors to give readers additional information about their work.

© 2021 Sjöbom U et al. *JAMA Network Open.*

eAppendix. Analytic Mesursements, Variable Definitions, Statistics, and GFAP Results

Analytical measurements

Samples for measurements were collected for included infants according to cohort-specific protocols. The samples were frozen and stored below -70ºC in adequately monitored freezers until the measurements. When the samples have been used for earlier measurements, they have been carefully thawed on ice for a minimal time before re-frozen.

Samples were assayed for NfL and GFAP according to the kit protocol using a 1:4 onboard dilution. Samples were assayed in singlicate due to the limited volumes available; samples with insufficient volumes were excluded. Internal controls, pooled samples of serum and plasma, and calibrators were assayed in duplicate to estimate the inter-assay and intra-assay variability. Internal controls at high and low levels were assayed before and after study samples in 24 different runs. The NfL assay had inter-assay repeatability of 8.1% at a low level (average 11 ng/L) and 8.3% at a higher level (average 49.4 ng/L), and a mean (range) intra-assay coefficient of variability (CV) of 6.6% (0.5-19.5%). The GFAP assay had inter-assay repeatability of 11.7% at a low level (97.9 ng/L) and 9.7% at a higher level (281.5 ng/L), with a mean (range) intra-assay CV of 5.7% (0.1-22.3%).

Interpolated values were used for results outside the calibration limits. For GFAP, we analyzed 1 026 available samples from 202 of the 221 included infants. Ten samples had measurements outside the calibration limit (>4 000 ng/L); for 2 of these samples, no interpolated values were obtained. They are interpreted in this study as 10 000 ng/L, which was above the highest interpolated value. For NfL, we analyzed 1 273 samples representing 209 infants; 1 sample had a value above the quantification limit (2 268 ng/L), and 5 samples had values below the quantification limit (<2.2 ng/L).

© 2021 Sjöbom U et al. *JAMA Network Open.*

Variable definitions

Longitudinal data on NfL and GFAP were categorized into subgroups based on the postnatal day (PND) of sampling described in the manuscript. 48 infants had more than one NfL value per period, and 34 infants had more than one GFAP value. We included a total of 1 225 samples for NfL and 992 for GFAP in the final analysis.

Statistics

Orthogonal Projections to Latent Structures (OPLS) modeling using SIMCA software version 15.0 (Umetrics AB, Umeå, Sweden), was used to investigate birth characteristics and morbidities associated with serum NfL AUC weeks 2-4 and GFAP AUC weeks 2-4. The AUCs for NfL and GFAP were log-transformed to meet a normal distribution. Permutation tests (n=999) were applied to validate the OPLS models. Unit variance scaling was used in all models. Variable importance for the projection (VIP) scores for the model with NfL AUC weeks 2-4 (n=155) are listed in **eTable 2**. The model included 1 predictive but no orthogonal component (R^2X=18.6% [goodness of fit in X], R^2Y =49.3%, and Q^2=46.2% [goodness of prediction], ANOVA *P*<.001, **eFigure 3**). When underlying variables explaining GFAP AUC weeks 2-4 were explored, it yielded a non-significant model (not shown).

Results GFAP

Concentrations of serum GFAP at birth (cord blood) and PND 1 were compared in a subpopulation (cohort 3). We found no correlation between cord blood levels and GA, **eFigure 6A**, Spearman correlation coefficient=-0.191, (95 % confidence interval (CI) -0.53-0.23; *P*=.330; n=28). The median GFAP concentration in cord blood was 261.4 (range 104.1-2433.7) ng/L (n=28) and significantly higher at PND 1; 580.8 ng/L, (range 148.1-9 504.8 ng/L; n=42; *P*=.001). Among infants with both cord blood and PND 1 samples (n=11), no significant difference in GFAP was observed between birth and PND 1 (*P*=.13), **eFigure 6B.**

© 2021 Sjöbom U et al. *JAMA Network Open.*

Longitudinal serum levels of GFAP from birth until 15 weeks PNA are illustrated in **eFigure 6C.** The longitudinal relationships between GFAP and GA for the first 12 weeks are shown in **eFigure 1B**

No significant relationships were observed between the AUC for GFAP and morbidities (data not shown), and no association was found with any BSID outcome **eTable 6**

© 2021 Sjöbom U et al. *JAMA Network Open.*

eTable 1. Binary Logistic Regression Model for ROP Outcome

Variable	OR	*P*	95% Wald CI for OR Lower	Upper
Ln NfL AUC PN weeks 2-4	4.79	<.001	2.17	10.56
GA at birth	0.64	.002	0.47	0.85
BW-SDS	0.67	.02	0.47	0.95
Vaginal delivery	5.22	.002	1.84	14.80
Apgar score < 7	1.92	.26	0.61	6.02

Classification of ROP (n=150).
Abbreviations: ROP: retinopathy of prematurity, OR: odds ratio, CI: confidence interval, Ln: natural logarithm, NfL: neurofilament light, AUC: area under the curve, PN: postnatal, , GA: gestational age, BW-SDS: birth weight standard deviation score.

© 2021 Sjöbom U et al. *JAMA Network Open.*

eTable 2. Associations Between Clinical Parameters and NfL

Variable importance for the projection (VIP) scores from an orthogonal projection to latent structures model (n=155) aimed to identify relationships between the NfL AUC during PN weeks 2 to 4 (Y) and clinical variables (X). Model parameters were R^2X=18.6%, R^2Y =49.3%, and Q^2=46.2%. VIP scores >1 indicate variables with important contributions to the model, i.e., high correlations with NfL AUC week 2-4.

Variable	VIP	SE
GA at birth, days	1.92	0.20
BW, g	1.78	0.23
Mechanical ventilation >7 days	1.77	0.39
Any ROP	1.76	0.23
PDA	1.63	0.27
Mechanical ventilation, days	1.61	0.54
BPD	1.25	0.43
Cohort 3	1.24	0.25
Parental nutrition (intervention in RCT)	1.06	0.50
Cohort 1	1.05	0.44
Apgar<7 at 5 min	1.04	0.36
IVH	1.02	0.54
NEC	0.87	1.37
Way of delivery	0.62	0.60
Twin	0.42	0.56
Mortality	0.41	1.04
Preeclampsia	0.37	0.50
Head circumference SD (birth up to PN day 7)	0.29	0.41
Prenatal steroids	0.27	0.48
Chorioamnionitis	0.23	0.51
Cohort 2	0.13	0.28
Sex	0.09	0.29
SGA at birth	0.08	0.42
BW-SDS	0.06	0.22

Abbreviations: NfL: neurofilament light, AUC: area under the curve, PN: postnatal, GA: gestational age, BW: birth weight, ROP: retinopathy of prematurity, PDA: Patent ductus arteriosus, BPD: bronchopulmonary dysplasia, RCT: randomized control trial, IVH: intraventricular hemorrhage, NEC: necrotizing enterocolitis, SD: standard deviation, SGA: small for gestational age, BW-SDS: birth weight standard deviation score.

© 2021 Sjöbom U et al. *JAMA Network Open.*

eTable 3. Variables With Significant Contribution to Any ROP

Univariate analysis variables for retinopathy of prematurity (ROP).

Variable	*P*-value
Way of delivery	<.001
GA at birth	<.001
BW	<.001
BW SDS	.02
Twin, n (%)	.10
Apgar 1 minute	.005
Study site	.004
Head circumference SDS (birth up to 7 PND)	<.001
NfL week 2	<.001
NfL week 3	<.001
NfL week 4	<.001
Mechanical ventilation >7 days	<.001
BPD	<.001
IVH	.001
NEC	.007
PDA	<.001
NfL AUC weeks 2 to 4	<.001
NfL AUC weeks 2 to 5	<.001
NfL AUC weeks 1 to 4	<.001

Abbreviations: GA: gestational age, BW: birth weight, SDS: standard deviation score, PND: postnatal days, NfL: neurofilament light, BPD: bronchopulmonary dysplasia, IVH: intraventricular hemorrhage, NEC: necrotizing enterocolitis, PDA: Patent ductus arteriosus, AUC: area under the curve. Analysis utilizing the Mann-Whitney U test.

© 2021 Sjöbom U et al. *JAMA Network Open.*

eTable 4. ROC Curve Analysis for ROP Outcome

Variable	Cut-off	*ROC AUC (95% CI)*	*Sensitivity % (95% CI)*	*Specificity % (95% CI)*	*PPV % (95% CI)*	*NPV % (95% CI)*
Binary logistic model	0.52[1]	0.89 (0.84-0.95)	85.4 (76.3-92.0)	78.7 (66.3-88.1)	85.4 (76.3-92.0)	78.7 (66.3-88.1)
Ln NfL AUC week 2-4	4.12[2]	0.85 (0.78-0.91)	83.7 (74.5-90.6)	72.6 (59.8-83.2)	81.9 (72.6-89.1)	75.0 (62.1-85.3)
GA at birth	26.5[3]	0.80 (0.74-0.86)	75.8 (67.3-83.0)	78.8 (68.6-87.0)	84.0 (75.8-90.2)	69.1 (58.9-78.1)
NfL level	33.5[4]	0.81 (0.76-0.86)	81.0 (71.9-88.2)	71.8 (65.9-86.3)	80.2 (71.1-87.5)	72.9 (60.9-82.8)

1= Predicted probabilities for ROP based on binary logistic regression model, included variables: Ln NfL AUC for PN weeks 2 to 4, GA, BWSDS, mode of delivery and Apgar score at 7 minutes (<7), (n=150)
2= Ln NfL AUC levels for PN weeks 2 to 4 [ng/L], (n=154)
3= GA in weeks, (n=209)
4= NfL [ng/L] cut off based on all single concentrations between PN week 2 to 4 (n=325). Classification of ROP using the cut-off was based on each infants highest NfL concentration between PN week 2 and 4 (n=171).

Abbreviations: ROC: Receiver operating characteristics, ROP: retinopathy of prematurity, AUC: area under the curve, CI: confidence interval, PPV: positive predictive value, NPV: Negative predictive value, Ln: natural logarithm, NfL: Neurofilament light chain, PN: postnatal, GA: gestational age, BWSDS: birth weight standard deviation score

© 2021 Sjöbom U et al. *JAMA Network Open.*

eTable 5. ROC Curve Analysis for BSID Outcome Below the Threshold at 2 Years of Age

Variable	Cut-off	*ROC AUC (95% CI)*	*Sensitivity % (95% CI)*	*Specificity % (95% CI)*	*PPV % (95% CI)*	*NPV % (95% CI)*
Binary logistic model	0.29[1]	0.80 (0.70-0.90)	82.1 (63.1-93.4)	69.8 (55.7-81.7)	59.0 (42.1-74.4)	88.1 (74.4-96.0)
NfL AUC week 2-4	133.3[2]	0.77 (0.66-0.88)	67.8 (47.7-84.1)	81.1 (68.0-90.6)	65.5 (45.7-82.1)	82.7 (69.7-91.8)

1= Predicted probabilities for BSID-outcome based on binary logistic regression model included variables: NfL AUC for PN weeks 2 to 4, GA at birth and sex (n=81)
2= NfL AUC for PN weeks 2 to 4 [ng/L], (n=81)

Abbreviations: ROC: Receiver operating characteristics, BSID: Bayley Scales of Infant Development, AUC: area under the curve, CI: confidence interval, PPV: positive predictive value, NPV: Negative predictive value, NfL: Neurofilament light chain, PN: postnatal, GA: gestational age

© 2021 Sjöbom U et al. *JAMA Network Open.*

eTable 6. Longitudinal GFAP Levels Summarized by AUC at Weeks 2 to 4 and Spearman Correlation to Outcome in BSID II and BSID III

	GFAP	
**Variable**	**Spearman correlation coefficient (95% CI)**	***P*-Value**
Psychomotor Developmental Index (BSID II)	-0.03 (-0.39-0.33)	.87
Mental Developmental Index (BSID II)	0.03 (-0.34-0.38)	.89
Motor Composite Score (BSID III)	-0.23 (-0.58-0.20)	.28
Cognitive Composite Score (BSID III)	0.09 (-0.31-0.46)	.67
Language Composite Score (BSID III)	0.20 (-0.21-0.56)	.32

Abbreviations: AUC: area under the curve, GFAP: glial fibrillary acidic protein, BSID: Bayley's Scales of Infant Development, CI: confidence interval

© 2021 Sjöbom U et al. *JAMA Network Open.*

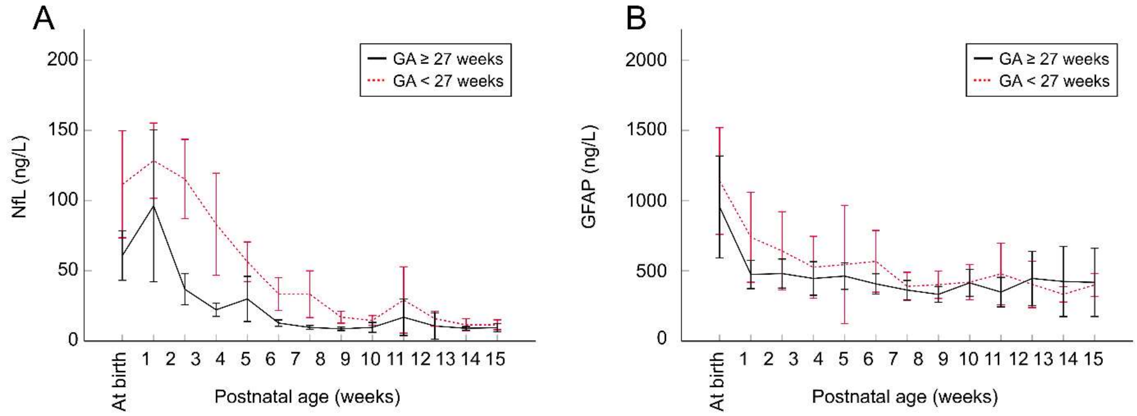

eFigure 1. Longitudinal Levels of NfL and GFAP From Birth Until 12 Postnatal Weeks

(**A**) Longitudinal levels of neurofilament light (NfL) in infants born before 27 weeks (n=145) of gestational age (GA) and after 27 weeks of GA (n=76). Infants born before 27 weeks of GA had significantly higher NfL levels between postnatal week 1-10 except for at week 2 and 5 (Mann Whitney U-test with Bonferroni-Holm correction). (**B**) Glial fibrillary acidic protein (GFAP) in 221 very preterm infants based on GA at birth before (n=145) or after 27 weeks (n=76). No significant difference was found between the groups.

© 2021 Sjöbom U et al. *JAMA Network Open.*

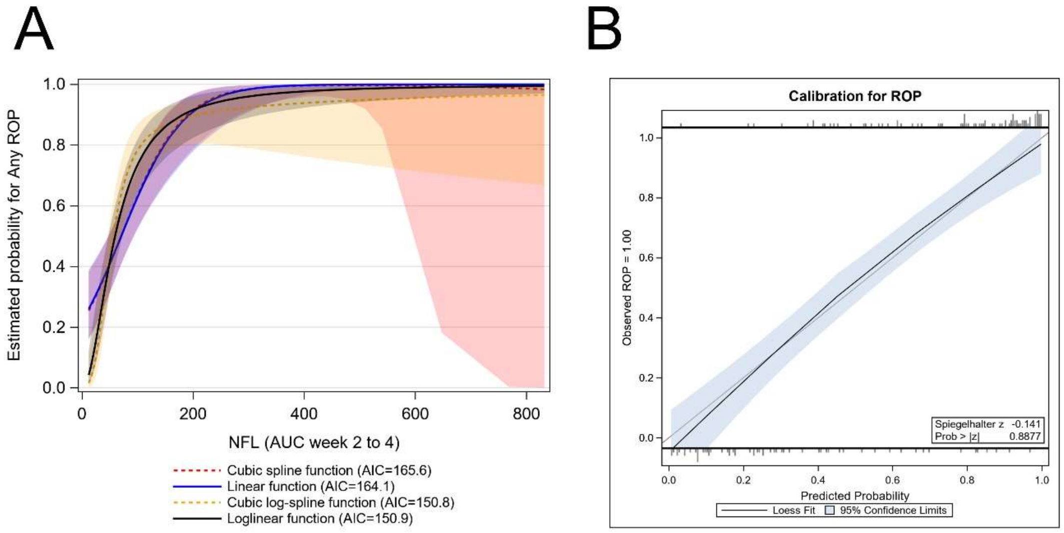

eFigure 2. Estimated Probabilities and Calibration Plot for Binary Logistic Regression Model ROP Outcome

(A) Different functions of longitudinal NfL levels, by area under the curve (AUC) between postnatal (PN) weeks 2-4, and their estimated probabilities for retinopathy of prematurity (ROP), 95% confidence intervals indicated by shaded area. The log-linear function was used for regression models based on the low Akaike information criterion (AIC) (AIC=150.9) **(B)** Calibration plot displaying observed proportions of ROP against predicted probabilities, based on binary logistic regression model. Included variables in ROP model: natural logarithm of NfL AUC for PN weeks 2 to 4, gestational age, birth weight standard deviation score, mode of delivery and Apgar score at 7 minutes (<7). Hosmer-Lemeshow test for goodness-of-fit, *P*=.56.

© 2021 Sjöbom U et al. *JAMA Network Open.*

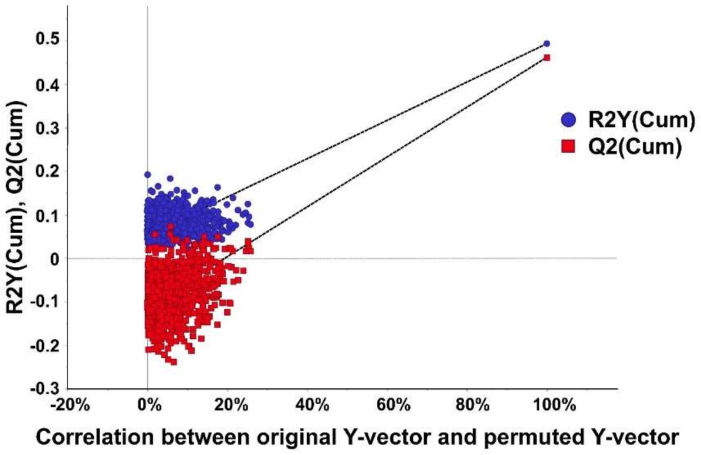

eFigure 3. Permutation Test for the OPLS Model

An orthogonal projection to latent structures (OPLS) model was used to identify variables associated with infant neurofilament light (NfL) levels between postnatal (PN) weeks 2 and 4 (n=155). The model included one predictive but no orthogonal component (R^2X=18.6% [goodness of fit in X]; R^2Y=49.3%; Q^2=46.2% [goodness of prediction]). The figure shows the results from a permutation test in which NfL levels were randomly permutated (n=999). The plot's vertical axis shows values for R^2Y and Q^2 in the permuted models (left) and the original model (far right). The horizontal axis shows the correlation coefficient between the permuted Y and the original Y. R^2Y and Q^2 values for all permutated models are lower than for the original model, indicating that the original model is strongly significant. The model was further validated using ANOVA of the cross-validated residuals (CV-ANOVA), where $p=4.9\times10^{-21}$.

© 2021 Sjöbom U et al. *JAMA Network Open.*

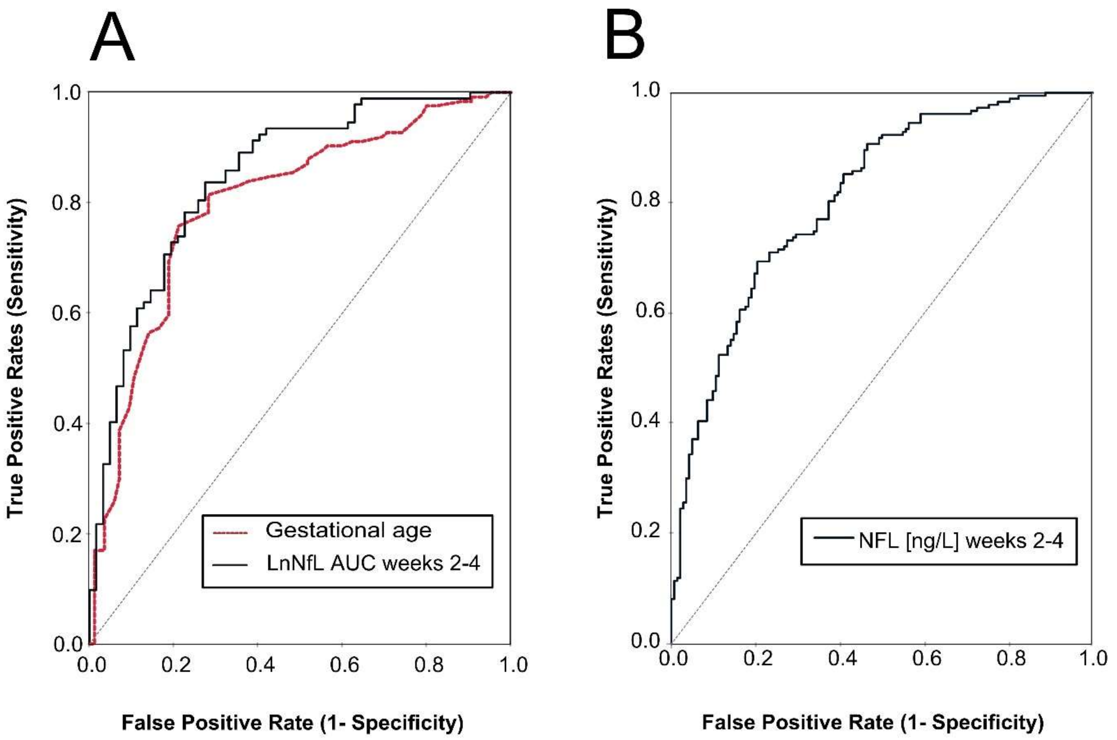

eFigure 4. ROC Curve Analysis for ROP Outcome

(A) The contribution of the natural logarithm of longitudinal Neurofilament Light (NfL) concentration summarized by area under the curve (AUC) between postnatal weeks 2-4 (n=154) and the association to retinopathy of prematurity (ROP) (ROC AUC 0.85) (black) compared to the association of gestational age (GA, red) and ROP (ROC AUC 0.8; n=209) in ROC analysis. **(B)** NfL concentrations between postnatal week 2 and 4 in ROC analysis to identify a single cut-off at 33.5 pg/mL with the highest explanatory to ROP outcome (ROC AUC 0.81, n=325 concentrations from 171 infants).

© 2021 Sjöbom U et al. *JAMA Network Open.*

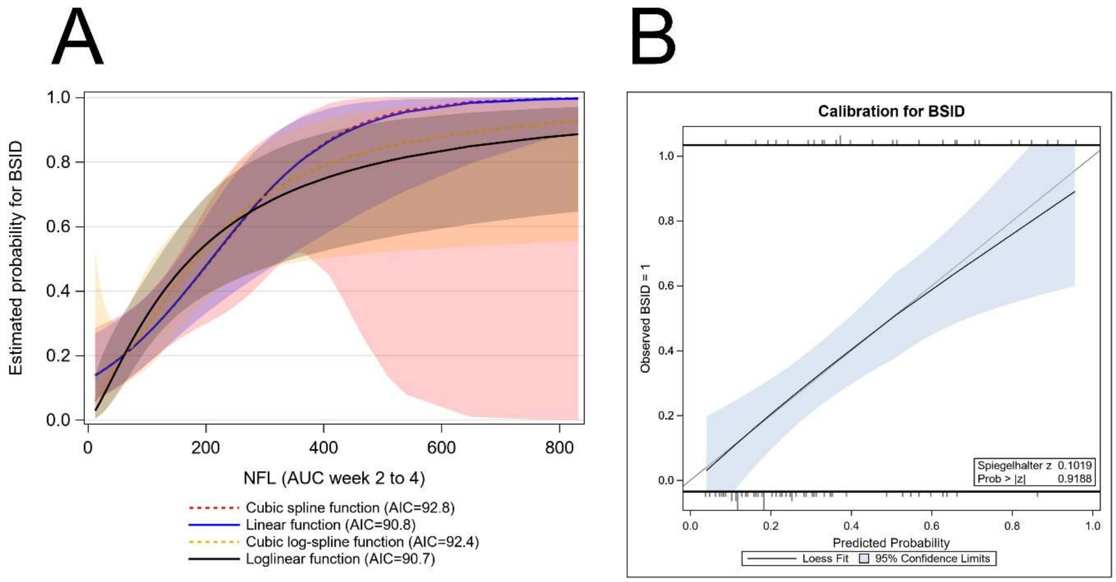

eFigure 5. Estimated Probabilities and Calibration Plot for Binary Logistic Regression Model BSID Outcome

(A) Different functions of longitudinal NfL levels, by area under the curve (AUC) between postnatal (PN) weeks 2-4, and their estimated probabilities, 95% confidence intervals indicated by shaded area, for poor Bayley's Scales of Infant Development (BSID) outcome. The linear function was used for regression models based on the low Akaike information criterion (AIC) (AIC=90.8) **(B)** Calibration plot displaying observed proportion of poor BSID outcome against predicted probabilities based on binary logistic regression model. Included variables in BSID model: NfL AUC for PN weeks 2 to 4, gestational age, and sex. Hosmer-Lemeshow test for goodness-of-fit, *P*=.87.

© 2021 Sjöbom U et al. *JAMA Network Open.*

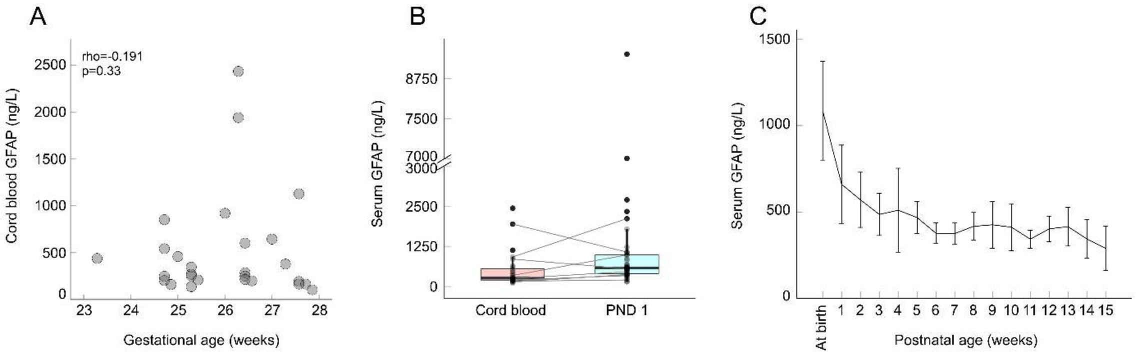

eFigure 6. Longitudinal Levels of GFAP From Birth Until 12 Postnatal Weeks

(**A**) Relationship between gestational age (GA) and glial fibrillary acidic protein (GFAP) in cord blood, no significant correlation was found (Spearman correlation coefficient=-0.191; 95 % confidence interval (CI) -0.53-0.23; *P*=.330; n=28). (**B**) GFAP distribution in cord blood (n=28) and on postnatal (PN) day 1 (n=42; Mann-Whitney U-test *P*=.001). We found no significant change for related samples (samples from one infant are connected with lines, Wilcoxon signed-rank test; *P*=0.13; n=11).

(**C**) Longitudinal levels of GFAP from birth until 15 postnatal weeks. GFAP was measured in 93, 94, 101, 64, 83, 77, 70, 78, 70, 57, 51, 48, 38, 37, 17, and 10 infants at birth and weeks 1-15, respectively. Error bars represent 95% CI.

© 2021 Sjöbom U et al. *JAMA Network Open.*
